# Data-Driven Method for Predicting Remaining Useful Life of Bearing Based on Bayesian Theory

**DOI:** 10.3390/s21010182

**Published:** 2020-12-29

**Authors:** Tianhong Gao, Yuxiong Li, Xianzhen Huang, Changli Wang

**Affiliations:** 1 School of Mechanical Engineering and Automation, Northeastern University, Shenyang 110819, China; gaotianhong960315@163.com (T.G.); 13354260226@163.com (Y.L.); a15698841933@163.com (C.W.); 2 Key Laboratory of Vibration and Control of Aero-Propulsion System Ministry of Education of China, Northeastern University, Shenyang 110819, China

**Keywords:** data-driven method, Bayesian model, Metropolis–Hastings algorithm, remaining useful life prediction

## Abstract

Bearings are some of the most critical industrial parts and are widely used in various types of mechanical equipment. Bearing health status can have a significant impact on the overall equipment performance, and bearing failures often cause serious economic losses and even casualties. Thus, estimating the remaining useful life (RUL) of bearings in real time is of utmost importance. This paper proposes a data-driven RUL prediction method for bearings based on Bayesian theory. First, time-domain features are extracted from the bearing vibration signal and data are fused to build a health indicator (HI) and a state model of bearing degradation. Then, according to Bayesian theory, a Bayesian model of state parameters and bearing life is established. The parameters of the Bayesian model are updated and bearing RUL is predicted by the Metropolis–Hastings algorithm. The method was validated by the XJTU-SY bearing open datasets and the prediction results are compared with the existing methods. Accuracy of the proposed method was demonstrated.

## 1. Introduction

Bearings are some of the most basic yet critical components used in the manufacturing industry and the overall performance and reliability of mechanical equipment are closely related to bearing performance [1]. Although bearings are the most commonly used components in mechanical equipment, bearings are also the most susceptible to failure [2]. Moreover, abnormal operating states can seriously affect production activities and may even lead to catastrophic consequences; therefore, predicting bearing remaining useful life (RUL) is of both theoretical and practical value. Since bearing RUL prediction is an important element of equipment prognostics and health management, extensive research has been carried out. Condition monitoring and RUL prediction of bearings in operation can help guide timely and reasonable maintenance, prolong service life of equipment, improve reliability of mechanical systems, and avoid catastrophic accidents caused by bearing damage [3,4].

Current methods for RUL prediction can be divided into two categories: model-based and data-driven methods. Model-based methods typically establish a degradation model according to the physical structure of the bearing, which is then used to predict the RUL of the bearing [5,6]. Jiang [7] proposed a prediction method for RUL based on the convex optimization-life parameter degradation mechanism model. Sun [8] established a Hertzian contact dynamic theory model of a bearing ball and raceway and showed that optimal damping can improve bearing life. Model-based methods require an accurate degradation model; however, complex structure of components and operation mechanisms, as well as environmental uncertainties in engineering practice, make it difficult to establish an accurate model [9]. Data-driven methods mainly rely on machine learning and deep learning algorithms to predict bearing RUL in the absence of a physical model of system degradation. This type of method can be universally applied for cases where the physical system cannot be accurately modeled. Numerous effective data-driven methods have been developed for RUL prediction. Wu [10] introduced long short-term memory (LSTM) networks to realize high-precision RUL prediction for complicated industrial objects. Ren [11] proposed a bearing RUL prediction method based on a deep neural network (DNN) and deep autoencoder. Xia [12] presented an innovative two-stage automated approach using a DNN to accurately determine the RUL of bearings. Li [13] constructed a modified health index based hierarchical gated recurrent unit network to improve the accuracy of bearing RUL prediction. Both supervised and unsupervised learning have also been applied in bearing fault diagnosis with good results [14,15,16,17].

Data-driven methods can overcome difficulties associated with model construction and can achieve more accurate prediction results. However, uncertainties still exist in practical applications, such as uncertainties in material properties, measurement errors, processing technologies, and operating conditions, which are often ignored. Owing to the high costs of product testing, and limitations of existing data transmission and storage technologies, sufficient data on the typical life cycle of bearings are usually unavailable. Therefore, in practice, data are insufficient to support data-driven prediction methods. Realizing a more accurate bearing RUL real-time prediction with limited bearing vibration signal data and considering the random factors is of great difficulty in related research. Bayesian theory is an effective method for data analysis with uncertain factors, which are regarded as random parameters. Expert knowledge, theoretical analyses, and historical data are used to obtain probability distributions of certain parameters (i.e., prior distributions). Then, updating methods are used to transform real-time data into more accurate distribution information (i.e., posterior distributions). Thus, the quantitative method of uncertainty based on the Bayesian theory has great research value in the field of RUL prediction. Mosallam [18] proposed a Bayesian approach for predicting the RUL of key components in systems with variable operating conditions. Cheng [19] presented a prediction method based on functional principal component analysis and the Bayesian method for Li-ion batteries RUL evaluation. Liu [20] proposed a dynamic data-driven layered Bayesian degradation model to tackle structural damage growth prediction. Tang [21] introduced a Bayesian Monte Carlo method to predict the aging trajectory of Li-ion batteries with significantly reduced experimental tests. Li [22] proposed a sequential Bayesian, which updated the Wiener process model improved the accuracy of RUL prediction. Martha [23] introduced a Bayesian hierarchical model to estimate the RUL of civil aerospace gas turbine engines.

**Figure 1:**
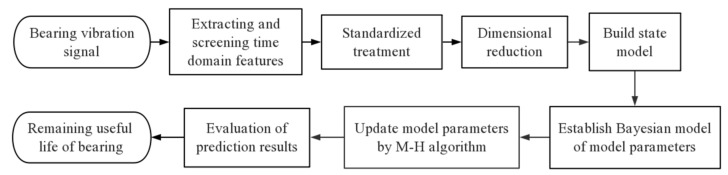
Flowchart of proposed bearing remaining useful life (RUL) prediction method.

This paper proposes a new data-driven bearing RUL prediction method based on Bayesian theory. A flowchart of the prediction process is illustrated in Figure 1. First, time-domain features are extracted from training bearing vibration signals and are screened. Standardization and dimensional reduction are carried out to build an appropriate health indicator (HI). Then, a state model of the bearing degradation process is established based on the processed data and a Bayesian model of state parameters and bearing life is constructed. Finally, parameters of the Bayesian model are updated according to real-time bearing data using the Metropolis–Hastings (M-H) algorithm to realize real-time prediction of bearing RUL.

## 2. Data-Driven State Model

### 2.1. Feature Index Selection

Time-domain indicators are commonly used in equipment fault detection and fault trend prediction, and can fully reflect the overall health degradation process of the system [24]. Common time-domain feature indexes include the mean value, root mean square value, peak value, and absolute mean amplitude. Considering the complex working conditions of bearings, a single feature index offers limited information for characterizing the bearing degradation process. Therefore, 16 time-domain feature indexes of bearing vibration signal data are extracted for the bearing RUL prediction. For details, see Table 1.

### 2.2. Data Fusion

Due to differences in the dimension and magnitude of each feature index, the multidimensional feature index data must be normalized. The Z-score standardization method is applied [25]:

(1) $$X_{i}^{*}:x_{i}^{*}\left(t\right)=\frac{x_{i}\left(t\right)-\mu_{i}}{\sigma_{i}}\;\;t=1,2,\cdots ,n$$

where $x_{i}\left(t\right),t=1,2,\cdots n$ is the time series of the *i*th dimensional feature index composed of *n* data, $X_{i}^{*}=\left(x_{i}^{*}\left(t\right),t=1,2,\cdots n\right)$ is the normalized *i*-dimensional time series data, and $\mu_{i}$ and $\sigma_{i}$ correspond to the mean and standard deviation of the *i*th dimensional time series data, respectively.

Although the multidimensional eigenvalue index contains sufficient information about the bearing degradation process, invalid information can be introduced resulting in increased computational complexity and reduced prediction efficiency. To overcome this, the multidimensional feature index data can be fused to construct a single bearing HI associated with the degradation process. Principal component analysis (PCA), which is one of the most widely used methods in data fusion, is applied [26,27]. The basic principle is to replace a large number of related variables with a small set of unrelated variables while retaining as much information as possible about the initial variables. Derived variables are called principal components, and are linear combinations of the initial variables. The basic steps of the PCA can be summarized as follows.

*n* groups of evaluation samples are set and each sample is evaluated by *m* indicators. The sample data can be expressed in the following form:

$$X=\left[\begin{array}{cccc} x_{11} & x_{12} & \cdots & x_{1m}\\ x_{21} & x_{22} & \cdots & x_{2m}\\ \vdots & \vdots & \vdots & \vdots \\ x_{n1} & x_{n2} & x_{n3} & x_{nm} \end{array}\right].$$

1. Calculate the correlation coefficient matrix of standardized data $X^{*}$. Then use the correlation coefficient to determine similarity among index variables.
2. Calculate the eigenvalues and eigenvectors of the correlation coefficient matrix. Eigenvalue $\lambda_{i}$ is the variance of the *i*th principal component $Y_{i}$. The eigenvector corresponding to each eigenvalue is a linear coefficient of variation, and the principal component $Y_{i}$ can be defined as
  (2) $$Y_{i}=\sum_{j=1}^{m}\alpha_{ij}X_{j}^{*}\;\;i=1,2,\cdots n$$
  where $Y_{i}$ is the *i*th principal component data (i.e., output data), $X_{j}^{*}$ is the *j*th dimensional original time series data (i.e., input data) after standardization, and $\alpha_{ij}$ is the linear transformation coefficient corresponding to the *i*th principal component and *j*th dimensional original time series data.
3. Calculate the variance contribution rate and cumulative contribution rate. The variance contribution rate reflects the role of index variables in the evaluation; the larger the value, the more effective the principal components are at retaining information. Generally, a principal component with an 85% cumulative contribution rate will meet calculation requirements.

The variance contribution rate of the *p*th principal component $Y_{p}$ can be expressed as

$$W_{p}=\lambda_{i}/\sum_{i=1}^{m}\lambda_{i}.$$

The cumulative contribution rate of principal components $Y_{1}$ $Y_{2}$ … $Y_{p}$ can be defined as

$$Z_{p}=\sum_{i=1}^{p}\lambda_{i}/\sum_{i=1}^{m}\lambda_{i}.$$

### 2.3. Establishment of HI and State Model

In this paper, the state model is established in the negative time scale based on two considerations: First, many studies assume the degree of degradation of the system is consistent at the initial time; however, due to complex working conditions and errors in manufacturing and assembly, the initial degradation of different systems will vary quite considerably. Second, many studies have only used system status monitoring data, whereas system life information is ignored. To improve the information utilization rate and facilitate prediction of the remaining life, bearing life in the negative time scale can be taken as one of the Bayesian model parameters. The negative time scale transformation formula is

(3) $$t_{i}^{*}=t_{i}-T_{i}\;\;0<i<n$$

where *n* is the number of training bearings, $t_{i}^{*}$ is time in the negative time scale, $t_{i}$ is time in the positive time scale, and $T_{i}$ is the system life. Thus, the state model in the negative timescale is

(4) $$H_{i}=F\left(t_{i}^{*},\theta_{i}\right)\;\;t^{*}\in \left[1-T_{i},0\right]$$

where $t^{*}\in \left[1-T_{i},0\right]$ is the negative time scale, with 0 representing bearing failure; $H_{i}$ is the HI of the bearing; $F\left(\cdot \right)$ is the state degradation model, which can be determined according to the trend of the maximum principal component over time; $\theta_{i}$ and are the state model parameters. By transforming the model into the negative time scale using Equation (3), the state model in the positive time scale can be obtained as

(5) $$H_{i}=F\left(t_{i}-T_{i},\theta_{i}\right)\;\;t\in \left[0,T_{i}\right]$$

## 3. Remaining Useful Life Prediction Model Based on Bayesian Theory

### 3.1. Bayesian Model

The bearing degradation process will be affected by uncertain factors, such as material properties, manufacturing and assembly processes, complex working conditions, and so on. In this paper, uncertainty in the prediction problem can be effectively dealt with by adopting the Bayesian model and probability method. There is a certain deviation between the bearing HI $H\left(t\right)$ and the measured value of HI $Y\left(t\right)$ (maximum principal component data), referred to as system noise. Noise usually obeys the standard normal distribution [1]. Therefore, the relationship between the measured value of $Y\left(t\right)$ and $H\left(t\right)$ is

(6) $$Y\left(t\right)=H\left(t\right)+\varepsilon$$

where $\varepsilon \sim N\left(0,\sigma^{2}\right)$ is noise with a standard normal distribution. According to the properties of a normal distribution, the measured value of $Y\left(t\right)$ satisfies the following normal distribution:

(7) $$Y\left(t\right)\sim N\left(H\left(t\right),\sigma^{2}\right).$$

Bayesian theory can be used to deduce the probability of unknown events based on the probability of known events [28]. The basic ideas behind Bayesian theory can be summarized as follows. An unknown parameter is regarded as a random variable. According to existing empirical information, a probability distribution of the variable, i.e., prior distribution, is obtained. The likelihood function is established and the distribution of variables is updated by fusing new information with existing prior distribution information to obtain a new posterior distribution. As the new data are gradually updated, the posterior distribution of the parameter will be closer to the real distribution. The process can be summarized as: the posterior distribution is proportional to the product of the prior distribution and the likelihood function. Considering uncertainty of bearing properties and randomness of service conditions, a Bayesian model of state parameters in the positive time scale can be established. The posterior distribution of state parameter θ and bearing life T can be expressed as

(8) $$f_{PIY}\left(\theta ,T|Y\left(t\right)\right)\propto f_{YIP}\left(Y\left(t\right)|\theta ,T\right)\cdot f_{P}\left(\theta ,T\right)$$

where $f_{YIP}\left(Y\left(t\right)|\theta ,T\right)$ is the likelihood function of the measured value of $Y\left(t\right)$ under state parameter θ and bearing life *T*; $f_{P}\left(\theta ,T\right)$ is the prior distribution of state parameter θ and bearing life *T*, which can be obtained from historical data on the bearing life cycle.

Since the vibration signal of the bearing is independently measured at each time point and the measured value $Y\left(t\right)$ of the HI is obtained by fusing multidimensional feature indexes extracted from the vibration signal, data are independent at each time for each value of HI. According to the nature of the independent variable, the total likelihood function is equal to the product of the likelihood functions at each time. From Equation (8), the posterior distribution of state parameters θ and system life *T* at the predicted time *k* can be obtained as

(9) $$f_{PIY}\left(\theta ,T|(Y\left(t\right)|t=1:k)\right)\propto \left(\prod_{t=1}^{k}f_{YIP}\left(Y\left(t\right)|\theta ,T\right)\right)\cdot f_{P}\left(\theta ,T\right).$$

### 3.2. Remaining Useful Life Prediction

To predict the RUL of the bearing at time *k*, parameters are updated according to the Bayesian model established in Equation (5) and measured HI values before time *k*. Then, the posterior distribution of model parameters $\left(\theta ,\;T\right)$ at time *k* is obtained. In general, calculating the posterior distribution of Bayesian model parameters is difficult. To solve this problem, the Markov chain Monte Carlo (MCMC) method can be applied to solve the posterior distribution [29].

The MCMC method is a sampling technique that can be used to extract samples from a probability density function (pdf). Posterior distribution samples are generated through stationary distribution of the Markov Chain and a Monte Carlo simulation is conducted. Here, the M-H algorithm is applied to calculate the posterior distribution [30]. The M-H algorithm constructs the proposal distribution $q\left(x\right)$ and generates samples, which are accepted or rejected according to a certain probability. Thus, a sample set conforming to the target distribution $p\left(x\right)$ is achieved. The specific steps of the algorithm are as follows:

1. Initialize starting point $x^{0}$.
2. For *N* − 1 iterations, complete the following four steps:
  - Draw a sample, x*, from the proposal distribution; the pdf value is $q\left(x^{*}|x^{i}\right)$ where *i* denotes the current iteration and the distribution mean is *x^i^* with a selected standard deviation.
  - Sample *u* from a uniform distribution with a lower limit of zero and an upper limit of 1, *U*(0,1).
  - Compute the acceptance ratio, $A=\min\left(1,\left(p\left(x^{*}\right)q\left(x^{i}|x^{*}\right)/p\left(x^{i}\right)q\left(x^{*}|x^{i}\right)\right)\right)$, where $q\left(x^{i}|x^{*}\right)$ is the pdf value of the proposal distribution at $x^{i}$ for the selected standard deviation, $p\left(x^{*}\right)$ is the pdf value of the target distribution at x*, and $p\left(x^{i}\right)$ is the pdf value of the target distribution at *x^i^*.
  - If *u* < *A*, set the new value of *x*, i.e., $x^{i+1}=x^{*}$. Otherwise, *x* remains unchanged, $x^{i+1}=x^{i}$.

In theory, any proposal distribution chain will gradually converge to the target distribution. Therefore, the M-H algorithm has good sampling effects for any target distribution. The selection of the proposed distribution affects the acceptance probability of the sample and convergence rate of the chain. In this paper, the proposal distribution is chosen as a uniform distribution based on empirical considerations and expert information. In addition, if the number of iterations is large enough, the initial value of the chain has no effect on the final sampling result [31]. Samples of the initial iteration are usually discarded as the training process and after the Markov chain is stable, the distribution can be taken as the sampling result.

## 4. Application of Proposed Method

### 4.1. Bearing Data

Datasets containing the complete run-to-failure data of 15 rolling element bearings (XJTU-SY) under accelerated degradation experiments were provided by the Institute of Design Science and Basic Component at Xi’an Jiaotong University (XJTU) and the Changxing Sumyoung Technology Co., Ltd., Zhejiang, China, (SY) [32]. For accelerated degradation experiments, a total of three different operating conditions were set and five bearings were tested under each operating condition. The sampling period was 1 min and the sampling frequency was set to 25.6 kHz. A total of 32,768 data points were recorded in 1.28 s during each sampling process. In our analysis, the bearing dataset of working condition 1 was selected for RUL prediction. Bearing 1_1 was taken as the test bearing and bearings 1_2, 1_3, 1_4, and 1_5 were selected as training bearings 1–4, respectively. Specific information extracted from the bearing datasets are shown in Table 2.

An HI and state model were established using signal data of the training bearings and prior information for the Bayesian model of state parameters and bearing life were obtained. Bearing 1_1 data were used as the test data to carry out the real-time RUL prediction. Finally, the prediction results were compared with the real RUL to verify the method.

### 4.2. Data Processing

**Figure 2:**
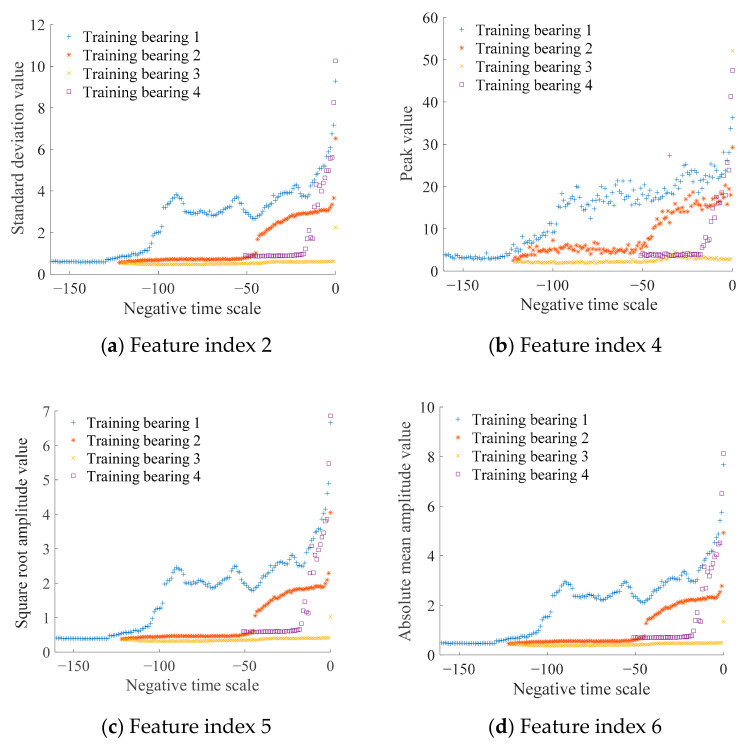

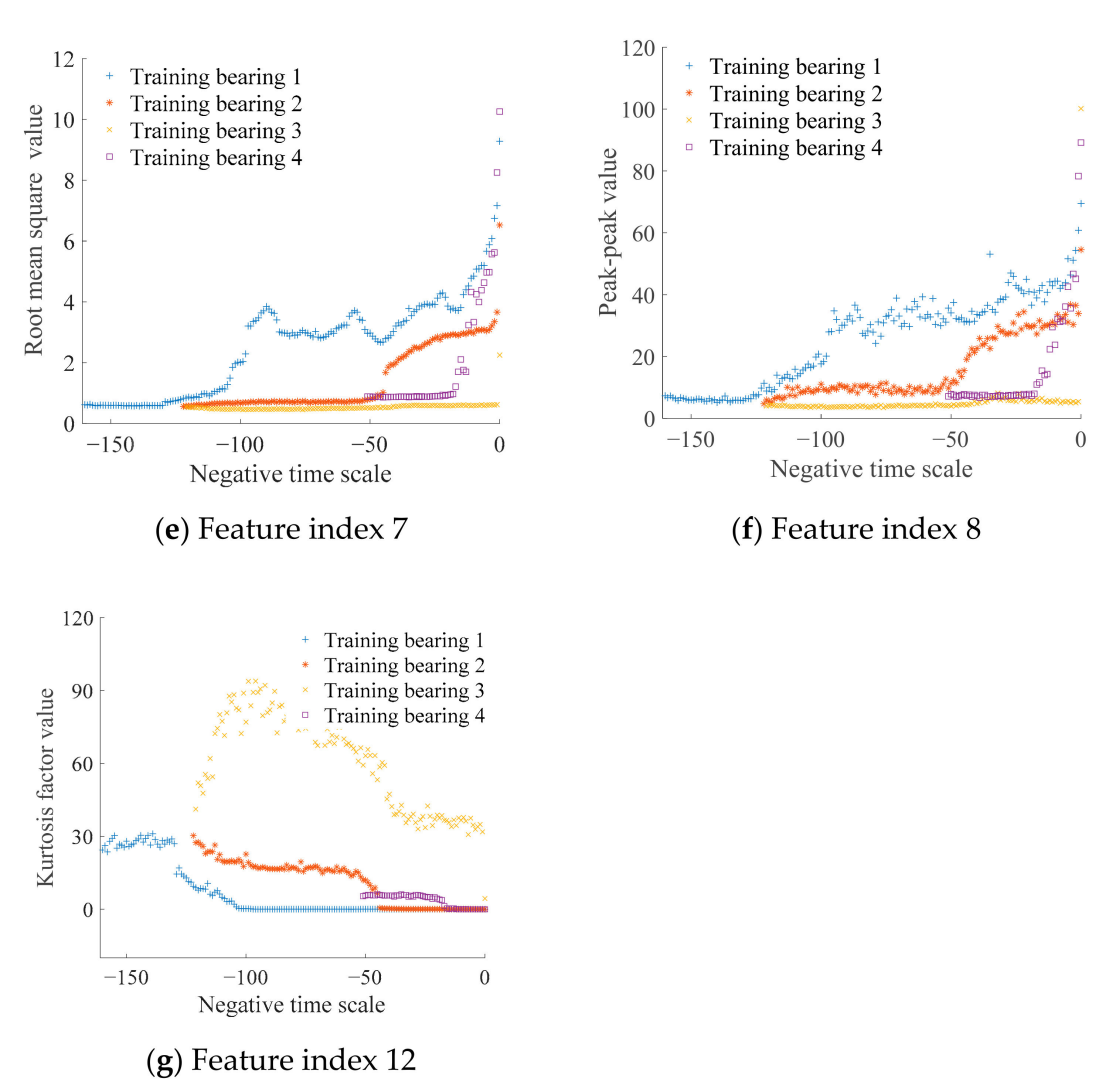
Data trends of selected feature index.

As listed in Table 1, 16 time-domain feature indexes of the four training datasets were extracted and plotted in the negative time scale to observe the degradation trends over time. To ensure the feature indexes accurately reflected the degradation process of the bearings, feature indexes 2, 4, 5, 6, 7, 8, and 12 with obvious degradation trends were selected for further analysis, as shown in Figure 2.

**Figure 3:**
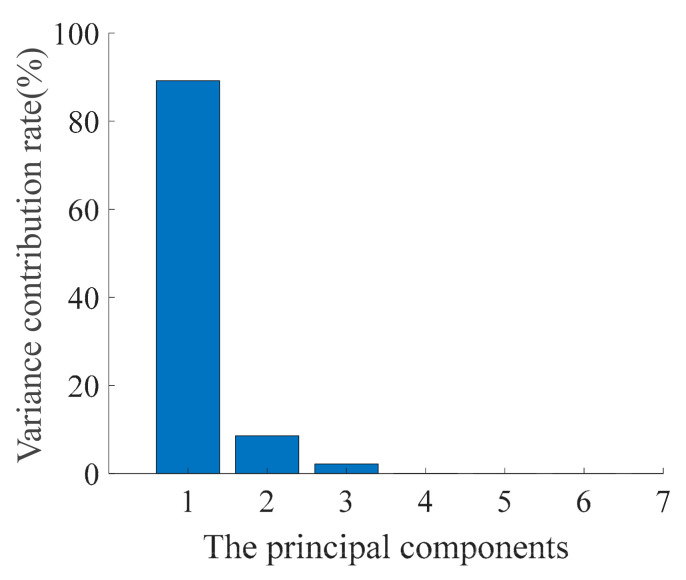
Variance contribution rates of each principal component.

**Figure 4:**
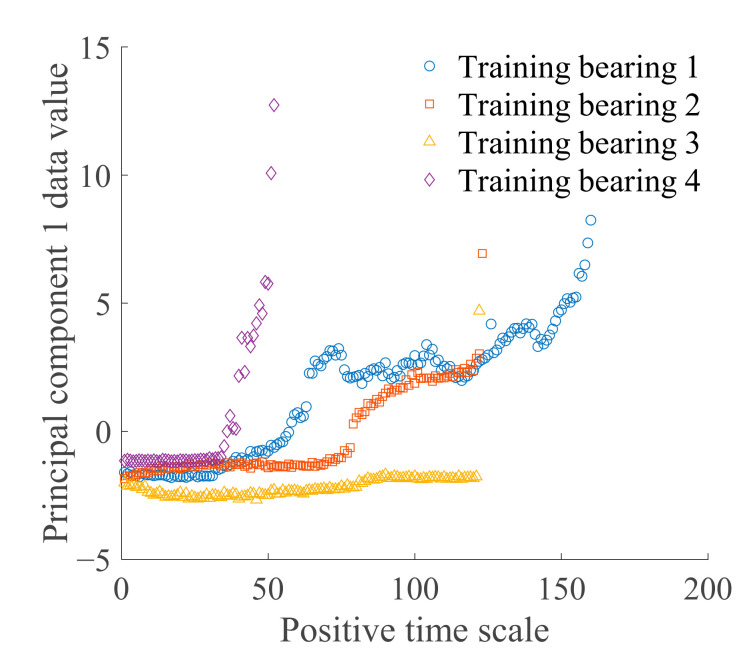
Principal component 1 data.

The seven selected feature indexes were standardized using Equation (1), and then the PCA method was applied to effectively achieve dimension reduction. Figure 3 shows the contribution rate of each principal component after data fusion. Principal component 1 retains sufficient information from the original data as the variance contribution rate of principal component 1 is up to 89.1969%. Therefore, principal component 1 was selected as the measurement value of the HI. Table 3 shows the corresponding linear transformation coefficients of 7 selected feature indexes in principal component 1. The principal component 1 can be calculated by introducing the linear transformation coefficient into Equation (2).

(10) $$Y_{1}=\sum_{j=1}^{7}\alpha_{1j}X_{j}^{*}$$

where $\alpha_{1j}$ represents the transformation coefficient of principal component 1 corresponding to the *j* selected feature indexes. Figure 4 shows the trends of principal component 1 of the four training bearings. Principal component 1 clearly changes over time, which can adequately reflect degradation of the bearing and further verifies the rationality of selecting principal component 1 to build HI and state model.

### 4.3. Establishment of HI and State Model

Selection of a reasonable model is the foundation of high-precision prediction. To reflect changes in the value of HI (principal component 1) over time (Figure 4), an exponential model was selected and used to establish the state model, expressed as

(11) $$H_{i}\left(t^{*}\right)=\exp[a_{i}\cdot t^{*}+b_{i}]+c_{i}\;\;t^{*}\in \left[1-T_{i},0\right]$$

where $H_{i}\left(t^{*}\right)$ is the HI and $a_{i}$, $b_{i}$, and $c_{i}$ are the state parameters of the *i*th training bearing. According to Equation (3), the state model can be expressed in the positive time scale as

(12) $$H_{i}\left(t\right)=\exp[a_{i}\cdot (t-T_{i})+b_{i}]+c_{i}\;\;t\in \left[1,T_{i}\right].$$

**Figure 5:**
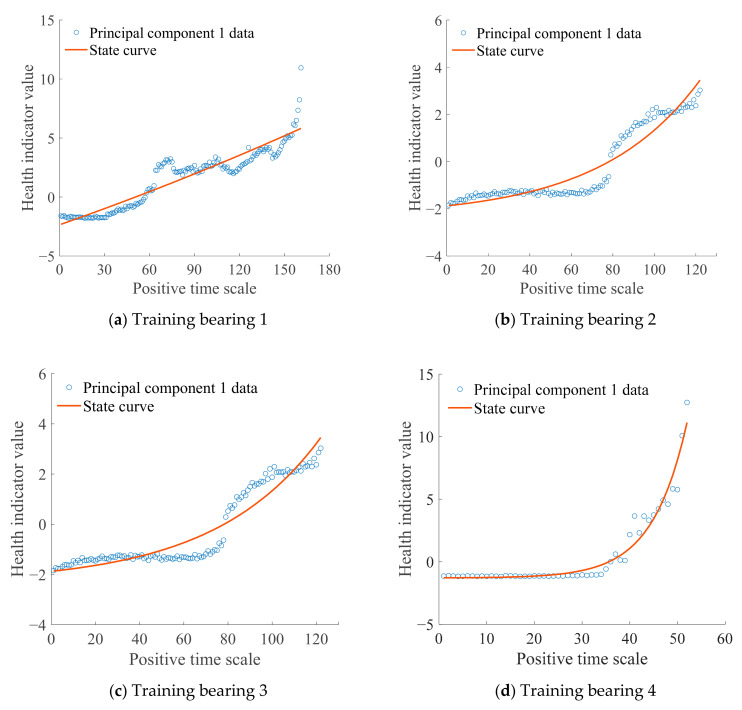
Training bearing state model.

Measured values of health indicators (HIs) and the bearing life of the training bearings were introduced into Equation (12) and the parameter estimation values of each training bearing state model were obtained using the least squares method. Figure 5 shows state curves of the four training bearings. The degradation process of training bearing 3 is stable until unexpected breakdown occurs at the last sampling point, which shows a lack of universality. Thus, data of training bearing 3 are not considered when building the Bayesian model. Differences can be observed in the degradation process and state parameters of training bearings 1, 2, and 4. Therefore, three prior samples of state parameters *a*, *b*, and *c* and bearing life *T* provide a suitable reference for the Bayesian model. In addition, the mean and variance of noise were obtained using statistics. Since noise is assumed to follow a normal distribution, the mean value of noise was zero and the three prior samples of noise variance were obtained.

### 4.4. Bayesian Model and RUL Prediction

The feature indexes screened in Section 4.2 were extracted from the test data and standardized using Equation (1). Based on the linear transformation coefficients presented in Table 3 and Equation (2), PCA dimensional reduction was carried out on the standardized feature index data. One-dimensional data obtained from the transformation were used as the measured values of $Y\left(t\right)$. A Bayesian model in the positive time scale was established using Equation (9) and Equation (12). Since the posterior distribution of parameters *a*, *b*, *c*, and *T* cannot be solved directly, the M-H algorithm was applied to perform independent sampling. For the test bearing, the prior distribution of parameters *a*, *b*, and *c* was set as a uniform distribution. According to three prior samples obtained from the historical data, the range of uniform distribution was determined to be $\left[\bar{a}-z,\bar{a}+z\right]$, where $\bar{a}$ is the mean value of three prior samples and *z* is a known constant used to describe differences among bearing degeneration. The prior distribution of *b* and *c* is determined in the same way. When the M-H algorithm was applied, the prior distribution of parameters *a*, *b*, and *c* were taken as the proposal distribution. The prior distribution of parameter *T* was set as a normal distribution. The mean value depends on the current value of the Monte Carlo chain and the standard deviation is 0.2. For example, the current value of the Monte Carlo chain is $T^{i}$ with prior distribution $N\left(T^{i},0.2\right)$ in the *i*th iteration. A candidate sample $T^{*}$ is randomly selected from the proposed distribution $N\left(T^{i},0.2\right)$. If the acceptance ratio *A* is greater than random variable *u*, sample $T^{*}$ is accepted with $T^{i+1}=T^{*}$; otherwise, $T^{i+1}=T^{i}$. Following the above method, the prior distribution of parameter *T* was selected as the normal random walk distribution and the prior distribution was taken as the proposal distribution of parameter *T* sampling.

**Figure 6:**
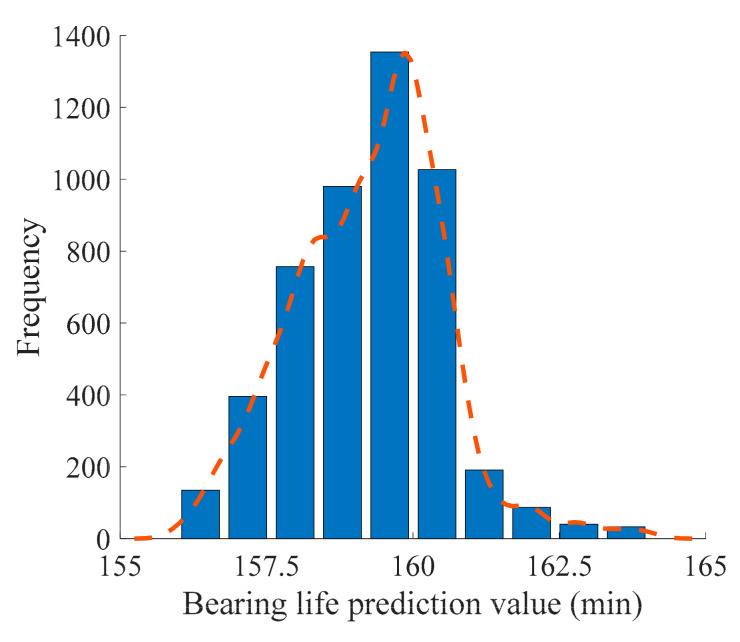
Posteriori distribution diagram of bearing predicted life (predicted time *k* = 110).

Considering the bearing is in the normal working state and runs smoothly in the early sampling stage, it is necessary to determine the starting point of bearing failure as the initial point of the RUL prediction, which better reflects the degradation trend of the bearing. The HI of the test data increases suddenly when the time series reaches 60 in the positive time scale; therefore, this point was taken as the starting point of bearing failure. Using the measured value of the test HI and the Bayesian model, the MCMC algorithm was applied to update the model parameters. A total of 10,000 iterations were used for each prediction. The first 5000 were used for the training process and the final 5000 update samples were taken as the posterior distribution samples of the model parameters. The posterior distribution of the predicted bearing life *T* was obtained, and the RUL posterior distribution was then obtained by subtracting the cut-off time of the test data. The expectation of the distribution sample was taken as the final prediction result. Figure 6 shows the distribution of the bearing life posteriori samples at the predicted time of 110.

**Figure 7:**
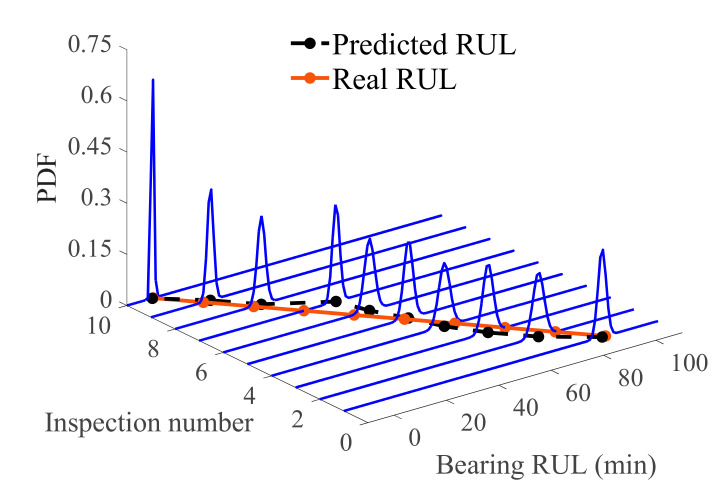
Probability density diagram of bearing RUL.

**Figure 8:**
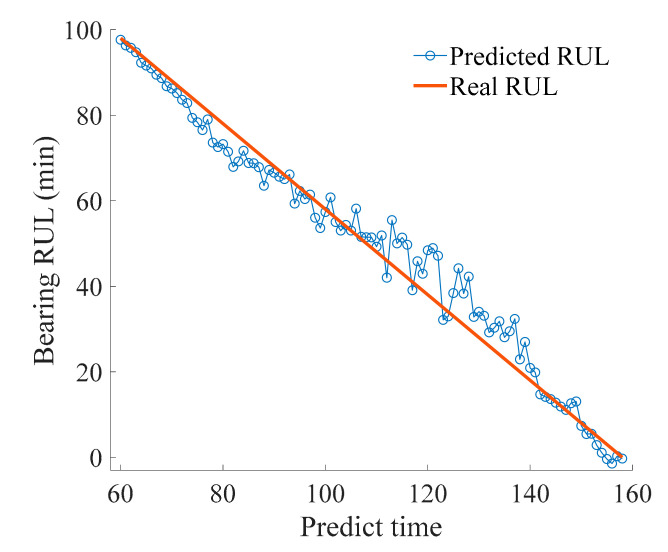
Comparison of predicted RUL and real RUL.

After the prediction update, the posterior distribution of bearing RUL is composed of 5000 samples. The probability distribution of the posterior sample is shown in Figure 7. Inspection numbers 1 to 10 correspond to the prediction time series 68, 78, 88, …, 158. The posteriori samples are represented by the probability density of the normal distribution and the expectation of each posteriori distribution is taken as the final prediction result. Comparison with the real bearing RUL demonstrates the accuracy of the proposed method. At the same time, the probability density distribution of the prediction results is relatively concentrated, indicating good stability. Moreover, the probability density curve tends to become more concentrated over time, indicating that the impact of uncertainty gradually decreases as the prediction progresses. A certain deviation exists between the predicted result and the actual value at the inspection number 7, which is related to the trend of the predicted bearing vibration signal data. The unstable, fluctuating trend of the original data causes deviation of the predicted result. Figure 8 shows a certain error in the prediction time between 110 and 140, which is caused by divergence of the measured values of the test HI that are used to update the Bayesian model. However, the subsequent prediction deviations gradually decrease, and prediction results are stable around the real RUL value, indicating that the overall prediction result is accurate.

### 4.5. Evaluation of Prediction Results

In order to quantitatively evaluate the prediction effect, three RUL prediction evaluation methods were adopted in this paper: root mean square error (RMSE), mean relative error (MARE) and error function based on asymmetric exponential [33,34].

RMSE denotes the root mean square of the prediction errors, which can be expressed as:

(13) $$RMSE=\sqrt{\frac{1}{N}\sum_{i=1}^{N}{\left(x_{i}-z_{i}\right)}^{2}}$$

where *x_i_* and *z_i_*, respectively, represent the predicted value and the real value of the *i*th prediction; *N* is the total number of real-time predictions. A smaller RMSE value means that RUL predicts better effectiveness.

MARE is the mean value of relative error among all time point. The expression of MARE is as follows:

(14) $$MARE=\frac{1}{N}\sum_{i=1}^{N}\left|\frac{x_{i}-z_{i}}{z_{i}}\right|\times 100\%.$$

Obviously, the approaches with smaller MARE would be better than others.

Error function based on asymmetric exponential can comprehensively evaluate the accuracy of the prediction method by constructing the exponential error between the predicted value and the true value and synthesizing the prediction accuracy of each prediction time series. According to RUL prediction results, the total evaluation error S can be calculated as follows:

(15) $$S=\sum_{i=1}^{N}S_{i},$$

(16) $$S_{i}=\left\{\begin{array}{l} \exp(-d_{i}/13)-1\;\;d_{i}\leq 0\\ \exp(d_{i}/10)-1\;\;d_{i}>0 \end{array}\right.,$$

(17) $$d_{i}=x_{i}-z_{i},$$

where *S* is the total evaluation error of *N* predictions; $d_{i}$ is the RUL estimation error of the *i*th prediction; $S_{i}$ is the evaluation error of the *i*th prediction. As the overall error evaluation value *S* decreases, the prediction accuracy increases.

**Figure 9:**
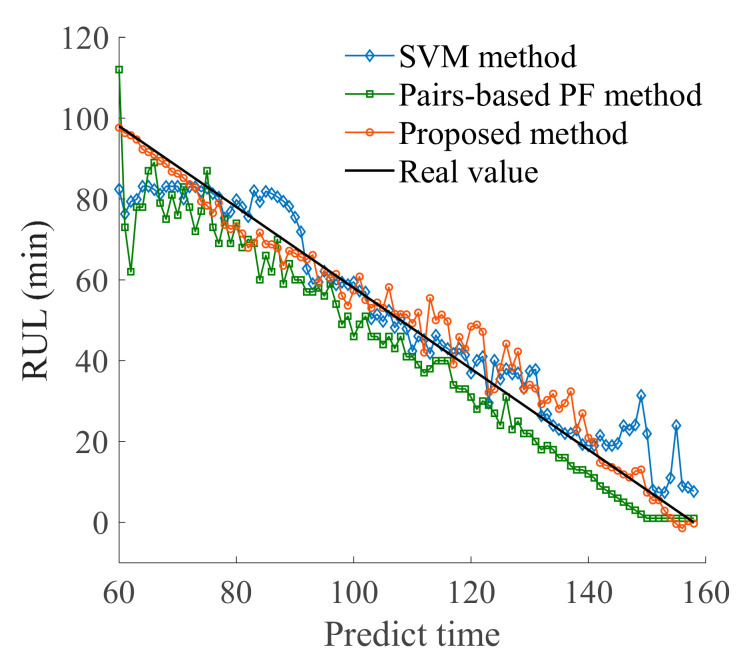
Comparison of prediction result.

**Figure 10:**
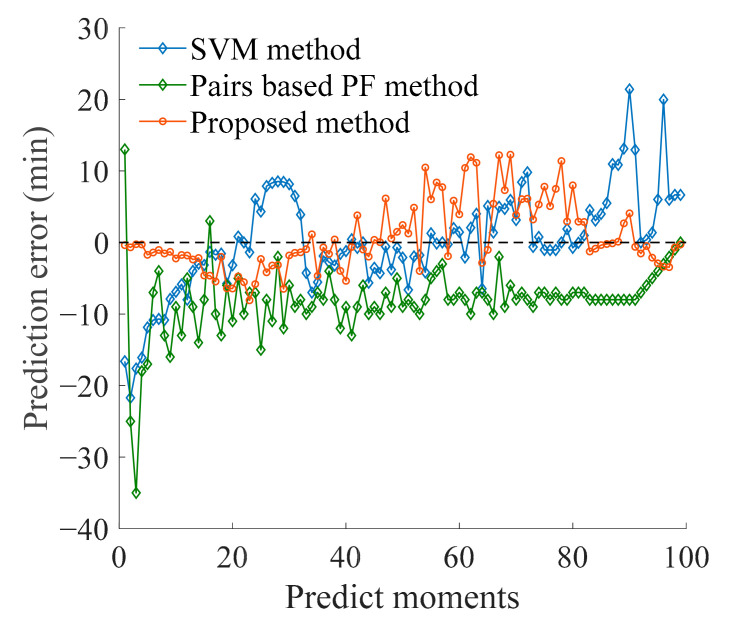
Comparison of prediction error.

The support vector machine (SVM) method and Pairs-based particle filter (PF) method were selected to illustrate the accuracy of the proposed method. The SVM is a widely used machine learning algorithm for classification and prediction, and can predict the RUL of bearings under small sample conditions with good prediction accuracy [35]. The Paris-based PF method combines physical model and observation data to identify model parameters, which is a model-based RUL prediction method [36]. Using the same bearing data and characteristic indexes as above, the SVM method and PF method were respectively introduced to predict the RUL of the bearing. The comparison of prediction results and errors is shown in Figure 9 and Figure 10, indicating that the method proposed in this paper has certain stability and accuracy. Then, the prediction errors of several methods are calculated according to Equations (13)–(17).Results of the analysis are presented in Table 4. The proposed method has good prediction accuracy and good stability.

## 5. Conclusions

This paper proposed a method of bearing RUL prediction based on Bayesian theory. Feature indexes reflecting the degradation trend were extracted from bearing vibration signals. The corresponding HIs were obtained through PCA and a state model was established. Information was extracted from limited historical data of samples to construct the prior distribution of the model parameters. A Bayesian model of state parameters was established and the MCMC algorithm was applied to update the Bayesian model parameters to obtain the posterior distribution of the RUL and its predicted value. The accuracy and stability of the method were verified using actual bearing data. In addition, the prediction results were compared with those obtained using an existing prediction method and the advantages of proposed method in terms of prediction accuracy were demonstrated.

Compared with other data-driven life prediction methods, the proposed method can be used to build a degradation model from limited existing data. Furthermore, the Bayesian approach effectively deals with parameter uncertainties in the degradation process. Therefore, prediction error is reduced and prediction accuracy and stability are greatly improved.

## Figures and Tables

**Table 1:** Selection of feature indexes.

Sequence Number	Feature Index	Expression	Sequence Number	Feature Index	Expression
1	Mean value	$\bar{X}=\frac{1}{N}\sum_{t=1}^{N}x_{i}$	2	Standard deviation	$X_{\sigma }=\sqrt{\frac{1}{N-1}\sum_{i=1}^{N}{\left(x_{i}-\bar{X}\right)}^{2}}$
3	Variation coefficient	$X_{cv}=X_{\sigma }/\bar{X}$	4	Peak value	$X_{\max}=\max\left\{\left|x_{i}\right|\right\}$
5	Square root amplitude	$X_{r}={\left[\frac{1}{N}\sum_{i=1}^{N}\sqrt{\left|x_{i}\right|}\right]}^{2}$	6	Absolute mean amplitude	${\bar{X}}_{p}=\frac{1}{N}\sum_{i=1}^{N}\left|x_{i}\right|$
7	Root mean square	$X_{rms}=\sqrt{\frac{1}{N}\sum_{i=1}^{N}x_{i}{}^{2}}$	8	Peak-to -peak	$X_{p-p}=\max\left(x_{i}\right)-\min\left(x_{i}\right)$
9	Skewness	$X_{ske}=\frac{\sum_{i=1}^{N}{\left(x_{i}-\bar{X}\right)}^{3}}{\left(N-1\right)X_{\sigma }^{3}}$	10	Kurtosis	$X_{kur}=\frac{\sum_{i=1}^{N}{\left(x_{i}-\bar{X}\right)}^{4}}{\left(N-1\right)X_{\sigma }^{4}}$
11	Skewness factor	$I_{ske}=\frac{X_{ske}}{X_{rms}^{3}}$	12	Kurtosis factor	$I_{kur}=\frac{X_{kur}}{X_{rms}^{4}}$
13	Crest factor	$I_{p}=\frac{X_{\max}}{X_{rms}}$	14	Impulse factor	$I_{i}=\frac{X_{\max}}{{\bar{X}}_{p}}$
15	Waveform factor	$I_{w}=\frac{X_{rms}}{X_{p}}$	16	Margin factor	$I_{m}=\frac{X_{\max}}{X_{r}}$

**Table 2:** Bearing datasets.

Operating Condition	Bearing Dataset	Number of Files	Bearing Lifetime	Fault Element
Condition1 (35 Hz/12 kN)	Bearing 1_1 (Test bearing)	158	2 h 38 min	Outer race
	Bearing 1_2 (Training bearing 1)	161	2 h 41 min	Outer race
	Bearing 1_3 (Training bearing 2)	123	2 h 3 min	Outer race
	Bearing 1_4 (Training bearing 3)	122	2 h 2 min	Cage
	Bearing 1_5 (Training bearing 4)	52	52 min	Inner and outer race

**Table 3:** Linear transformation coefficients of principal component 1.

Selected Feature Index	$X_{\sigma }$	$X_{\max}$	$X_{r}$	${\bar{X}}_{p}$	$X_{rms}$	$X_{p-p}$	$X_{kur}$
Coefficient value $\alpha_{1j}$	0.3960	0.3940	0.3947	0.3959	0.3959	0.3859	−0.2692

**Table 4:** Comparison of prediction results.

Prediction Method	SVM Method	Pairs Based PF Method	Proposed Method
RMSE	7.1005	8.7427	4.8843
MARE	3.2405	4.7628	2.3079
S value	80.7027	94.8183	46.5061

## Data Availability

Data sharing not applicable.
